# Alignment of time course gene expression data and the classification of developmentally driven genes with hidden Markov models

**DOI:** 10.1186/s12859-015-0634-9

**Published:** 2015-06-18

**Authors:** Sean Robinson, Garique Glonek, Inge Koch, Mark Thomas, Christopher Davies

**Affiliations:** 1 School of Mathematical Sciences, University of Adelaide, Adelaide, Australia; 2 CSIRO Agriculture, Adelaide, Australia

**Keywords:** Alignment, Classification, Hidden Markov models, Time course microarray experiment

## Abstract

**Background:**

We consider data from a time course microarray experiment that was conducted on grapevines over the development cycle of the grape berries at two different vineyards in South Australia. Although the underlying biological process of berry development is the same at both vineyards, there are differences in the timing of the development due to local conditions. We aim to align the data from the two vineyards to enable an integrated analysis of the gene expression and use the alignment of the expression profiles to classify likely developmental function.

**Results:**

We present a novel alignment method based on hidden Markov models (HMMs) and use the method to align the motivating grapevine data. We show that our alignment method is robust against subsets of profiles that are not suitable for alignment, investigate alignment diagnostics under the model and demonstrate the classification of developmentally driven genes.

**Conclusions:**

The classification of developmentally driven genes both validates that the alignment we obtain is meaningful and also gives new evidence that can be used to identify the role of genes with unknown function. Using our alignment methodology, we find at least 1279 grapevine probe sets with no current annotated function that are likely to be controlled in a developmental manner.

**Electronic supplementary material:**

The online version of this article (doi:10.1186/s12859-015-0634-9) contains supplementary material, which is available to authorized users.

## Background

Alignment of time course gene expression data is an important problem since, ‘biological processes have the property that multiple instances of a single process may unfold at different and possibly non-uniform rates in different organisms, strains, individuals, or conditions’ [1]. Such different rates may affect the timing of gene expression, which will be manifest in the observed expression profiles.

**Fig. 1:**
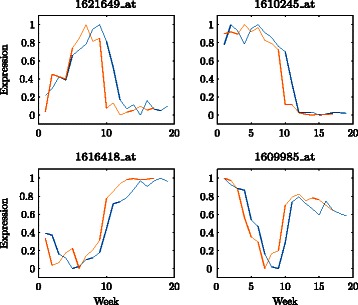
Expression profiles for four example genes from the Willunga (blue) and Clare (orange) vineyards

We consider a time course microarray experiment conducted on grapevines (*Vitis vinifera* L., Cabernet Sauvignon) at the ‘Willunga’ and ‘Clare’ vineyards in South Australia. The experiment was run over the duration of the development cycle of the grape berries, from the closed-flower to ripe-red stage of the berries themselves. For each gene, we have a pair of expression profiles, one from each of the Willunga and Clare vineyards. Pairs of expression profiles for four example genes can be seen in Fig. 1. For each pair of profiles, we aim to obtain a single profile that captures the relevant gene expression information over the development cycle of the grape berries from both vineyards. The common representations can then be used for an overall analysis of the gene expression.

The rate of development of the grape berries was different at the Willunga and Clare vineyards. Differences between the vineyards such as soil conditions, viticultural management and climate are likely causes of the different rates of berry development [2]. During the experiment, the length of the development cycle was 19 weeks at Willunga and 17 weeks at Clare. Since the experiment called for weekly measurements, the expression profiles from Willunga have length 19 while the expression profiles from Clare have length 17 (Fig. 1). Hence we require an alignment between the different length profiles.

The basic underlying pattern of berry growth and ripening was the same at both the Willunga and Clare vineyards, which suggests a common underlying framework of gene expression control. Hence in spite of the different conditions, if a pair of expression profiles exhibit the same basic shape at both vineyards and are suitable for alignment, this is strong evidence that the corresponding gene is likely to be developmentally controlled. On the other hand, pairs of profiles with different shapes are not suitable for alignment and the corresponding gene is unlikely to be driven by the development process but by other factors.

A recent survey of grapevine genes [3] indicated that the annotation of 44 % of genes is ‘poorly informative’ (including 29 % having no Blast hit and 9 % with function unknown). Actual functional data is available for only a small subset of those genes with an assigned function and most often function is defined on the basis of sequence similarity with genes from other species. Additionally, the assignment of a biochemical function does not define whether a gene has a mainly developmental role or is merely responding to external cues.

Hence considering whether a pair of profiles is well aligned will give important additional evidence that can be used to identify genes as either likely to be developmentally driven or not.

The time sparsity and variability of the grapevine data is typical of longer term time course gene expression experiments. Interpolation of the expression values between observed time points is not readily justified as significant non-linear variations in expression could conceivably occur between adjacent time points. Rather than the expression levels week by week, the biological relevance is in the general expression behaviour over the entire development cycle, which is where both the available data and current biological understanding lie.

Non-model based alignment methods such as discrete time warping (DTW) have been used for alignment of time course gene expression data [1]. However, for the grapevine data, DTW invariably produces pathological results. For example, >3 time points mapped to a single time point from Willunga to Clare immediately followed by the same from Clare to Willunga has no reasonable interpretation when each time step is a week and especially when the alignment differs for different pairs of profiles. Simply considering the lag between profiles would also not be a suitable model for the timing differences between vineyards and would violate the experimental set-up.

In order to work with the typical sparsity of the grapevine data, as well as to provide a principled way to obtain a common alignment across both vineyards, we turn to hidden Markov model (HMM) based alignment methods.

### Left-right HMMs

Lin *et al.* [4] aligned gene expression profiles using an HMM by constraining the Markov chain component to be a ‘left-right’ model. In a left-right HMM a state can never be revisited once it has been left and transitions away from a state may only occur to a single other state. Hence an alignment is achieved between the expression profiles by considering the different times the state transitions occur in the corresponding Viterbi paths.

A left-right HMM can be altered to allow for less restrictive transitions between states while keeping the same alignment idea, for example allowing the ‘leapfrogging’ of states. Schliep *et al.* [5] considered such an alignment, however their main focus was a model-based ‘soft’ clustering method for expression profiles using mixtures of HMMs.

We aim to capture the basic pattern of each pair of profiles, which may be different from any other pair (Fig. 1). Hence approaches that constrain the Markov state transitions to the extent that all realised state sequences must share the same basic shape are not suitable in this case.

### Pair HMMs

Pair HMMs are the standard model for the alignment of genomic sequence data [6]. However, Pair HMMs require discrete emission random variables to model the genomic sequences of interest. In addition, the conditional information of a previous emission observation is not the actual observed value but whether the observation was a pair or single nucleotide symbol. Since we aim to interpret the underlying Markov structure as capturing distinct quantitative levels of the expression profiles, we require more than the binary pair/single nucleotide symbol dynamics of the Markov chain component of a Pair HMM.

### Extensions of Pair HMMs

Two ways in which Pair HMMs could be extended to model time course gene expression data are to:

- Retain the binary dynamics of the Markov chain component of the model and consider continuous emission random variables; or
- Incorporate additional information into the model so that the Markov structure encodes more than just binary dynamics.

Note that these possible extensions do not explicitly take alignment into account, although the motivation in considering such extensions is that the established alignment method of Pair HMMs could be carried over.

#### Binary Markov dynamics with continuous emissions

Yuan and Kendziorski [7], and Yoneya and Mamitsuka [8] both proposed extensions of a Pair HMM that retain the binary dynamics of the Markov chain component of the model. Both modelled time course gene expression data and hence considered continuous emission random variables. Yuan and Kendziorski [7] did not aim to obtain an alignment between expression profiles, and it is not clear how their model could be adapted for this purpose. Although the model of Yoneya and Mamitsuka [8] could be used as the basis of an alignment, their model requires strict assumptions about the shape of the expression profiles, assuming average expression levels except for at least one spike feature. Most genes in the grapevine data do not display expression profiles with such patterns (Fig. 1) so this approach is not suitable.

#### Additional information incorporated into the model

Listgarten *et al.* [9] proposed a ‘Continuous Profile Model’ (CPM), which they consider to be a ‘continuous analogue’ to a Profile HMM. Also widely used for the alignment of genomic sequence data, Profile HMMs are closely related to Pair HMMs [6]. Under a CPM, each time series is modelled as an emission sequence and the corresponding realisation of the state sequence is a mapping to an additional input sequence or ‘latent trace’. The latent trace has a higher number of time points than the observed time series (approximately double), which allows the mapping to ‘slow down’ and ‘speed up’ relative to ‘latent time’ and hence constitute an alignment.

The CPM was developed for mass spectrometry and speech waveform time series that were sampled frequently enough in time that interpolating smoothly between time points was a reasonable approach. The assumption of smoothness in time necessary for the ‘continuous’ CPM alignment is not reasonable for the grapevine data. Therefore, it would not be appropriate to apply the CPM alignment method to the grapevine data.

### Our approach

We will model the expression profiles as multiple emission sequences of an HMM so that each pair corresponds to a common underlying state sequence. The emission sequences are aligned under the model in that aligned emission random variables are conditioned by the same state random variable. We will assume that the underlying Markov state sequence represents a common expression profile at both vineyards and that the Markov states represent distinct quantitative levels of gene expression.

Like the CPM, our alignment HMM is conceptually similar to a Pair HMM. However, in contrast to Pair HMMs, the alignment in our model is not determined by the underlying Markov chain but through ‘gap position’ parameters, which we incorporate into the model as additional information. Rather than the latent trace and continuous time warping of Listgarten *et al.* [9], this coarse approach to alignment is necessitated by the sparsity of our data.

We use our alignment HMM to achieve an alignment of the grapevine data and quantify how well each pair of profiles is aligned. We show that our method of training the model is computationally efficient and also robust against subsets of profiles that do not align. We then consider diagnostics under the model and demonstrate that genes can be classified as either likely to be developmentally driven or not by how well they align.

## Methods

### Grapevine data

In addition to being from spatially distinct vineyards, the time course microarray experiment was run in the 2004 grape growing season at Willunga and in the 2005 grape growing season at Clare. Gene expression levels were measured weekly at both vineyards using Affymetrix grapevine GeneChips (Santa Clara, CA, USA, Part #520054). We discard the expression profiles not differentially expressed in time at the 0.001 % significance level using LIMMA [10], as well as those without at least a 2-fold change in expression level. We also discard all profiles corresponding to the *Vitis vinifera* Array (non vinifera / non 3 prime) Mask. We average the replicate expression observations at each time point and then linearly scale each profile individually so that all observed expression levels lie in the interval [0,1] (Additional file 1: Figure S1). We refer to the resultant 8644 pairs of profiles as the ‘grapevine data’.

### Alignment model

**Fig. 2:**
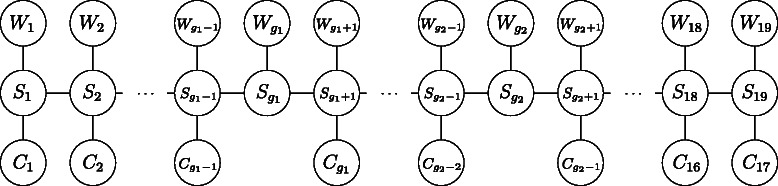
Conditional independence graph of the alignment HMM for the grapevine data

We present our alignment methodology based on an HMM for the scaled time course gene expression grapevine data. The conditional independence graph of the alignment model is given in Fig. 2. Each pair of expression profiles is modelled as the two sequences of emission random variables *W*_1:19_ and *C*_1:17_ (indexed by time) for the Willunga and Clare vineyards respectively. The alignment is obtained based on the assumption that both emission sequences arise from a single state sequence *S*_1:19_. The time points for the Willunga sequence *W*_1:19_ correspond directly to those of the common state sequence *S*_1:19_, while the time points for the Clare sequence *C*_1:17_ are obtained via ‘gap positions’ 1<*g*_1_<*g*_2_≤19. In our approach the gap positions are treated as parameters of the model to be estimated from the data.

For a single pair of expression profiles, there is usually insufficient information to identify optimal gap positions. However, since the grapevine data have been scaled so that all observed expression levels lie in the interval [0,1], the Markov state space and conditional emission distributions can be considered common for all genes. This allows us to estimate a single set of gap positions by pooling the data from all pairs of profiles.

The state random variables *S*_1:19_ that form the Markov chain component of the alignment HMM are discrete valued and take values in a common state space *Ω*_*S*_={1,2,…,*N*}. For convenience we use *p*(*x*) to symbolise both a probability density function and a probability mass function, in addition to using the event ‘ *X*=*x*’ as an argument.

Let *a*=(*a*_1_,*a*_2_,…,*a*_*N*_)^T^ be the *N*×1 vector of initial state probabilities and *A*={*a*_*ij*_} be the *N*×*N* state transition matrix of the Markov chain state sequence where

$$\begin{array}{*{20}l} a_{i} = p(S_{1} = i) \end{array} $$

for *i*=1,2,…,*N* and

$$\begin{array}{*{20}l} a_{ij} = p(S_{t} = j | S_{t-1} = i) \end{array} $$

for *i*,*j*=1,2,…,*N*.

Let $B = \left \{\mu _{1},{\sigma _{1}^{2}},\mu _{2},{\sigma _{2}^{2}},\dots,\mu _{N},{\sigma _{N}^{2}}\right \}$ be the set of all parameters of the Gaussian emission distributions so that

$$\begin{array}{*{20}l} p\left(x|S_{t} = j\right) = b\left(x|\mu_{j}, {\sigma^{2}_{j}}\right) \end{array} $$

where

$$\begin{array}{*{20}l} b\left(x|\mu_{j}, {\sigma^{2}_{j}}\right) = \frac{1}{\sqrt{2{\pi\sigma^{2}_{j}}}}\exp\Big\{-\frac{1}{2{\sigma_{j}^{2}}}(x-\mu_{j})^{2}\Big\} \end{array} $$

for *j*=1,2,…,*N*.

In the general case for the *k* ^*th*^ gene, we consider an underlying state sequence,

$$S^{(k)}_{1},S^{(k)}_{2},\ldots,S^{(k)}_{T} $$

and model the *L* expression profiles for each gene as the emission sequences

$$X^{(k)}_{l,1},X^{(k)}_{l,2},\dots,X^{(k)}_{l,T_{l}} $$

where *T*_*l*_≤*T* for *l*=1,2,…,*L*. The alignment of the *l* ^*th*^ expression sequence to the underlying common state sequence is defined by values

$$1\leq \tau_{l,1}<\tau_{l,2}<\cdots<\tau_{l,T_{l}}\leq T $$

that indicate the state positions corresponding to each observed expression value.

Taking the set of HMM parameters to be

$$\begin{array}{*{20}l} \lambda \equiv \{a,A,B\} \end{array} $$

and the set of alignments to be

$$\begin{array}{*{20}l} \boldsymbol{\tau}=\{ \tau_{l,t} \ | \ l=1,2,\dots,L\ \, \text{and }\ \, t=1,2,\dots, T_{l}\} \end{array} $$

the general alignment HMM log-likelihood can be written as

$${\small{\begin{aligned} \ell(\lambda, \boldsymbol{\tau} |\boldsymbol{x}) &=\sum_{k=1}^{K}\log\left[\sum_{(s_{1},s_{2},\ldots,s_{T})} p(s_{1},s_{2},\ldots,s_{T})\right.\\ &\quad \times\left.\prod_{l=1}^{L}\prod_{t=1}^{T_{l}}p\left(x^{(k)}_{l,t}\big|S_{\tau_{l,t}}=s_{\tau_{l,t}}\right)\right] \end{aligned}}} $$

where

$$p(s_{1},s_{2},\ldots,s_{T})=a_{s_{1}}\prod_{t=2}^{T}a_{s_{t-1}s_{t}} $$

and

$$p\left(x^{(k)}_{l,t}\big|S_{\tau_{l,t}}=s_{\tau_{l,t}}\right)= b\left(x^{(k)}_{l,t}\big|\mu_{s_{\tau_{l,t}}},\sigma^{2}_{s_{\tau_{l,t}}}\right). $$

The alignment is determined by maximising *ℓ* with respect to the HMM parameters *λ* and the alignment points ***τ***. The model underlying this likelihood allows each gene its own unique state sequence but imposes a common alignment over all genes.

For the grapevine data, *K*=8644, *L*=2 and *T*=19. Taking ***w*** and ***c*** to represent the expression data from Willunga and Clare respectively ($x^{(k)}_{1,t} = w^{(k)}_{t}$ and $x^{(k)}_{2,t} = c^{(k)}_{t}$ for *k*=1,2,…,8644 and *t*=1,2,…,*T*_*l*_), we have *T*_1_=19, *T*_2_=17 and

$$\tau_{1,t}=t\ \, \mathrm{for }\ \, t=1,2,\ldots,19. $$

The alignment is then determined by choosing

$$1= \tau_{2,1}<\tau_{2,2}<\cdots<\tau_{2,17}\leq19 $$

which can be specified by equivalently choosing two gap positions *g*_1_<*g*_2_ in the sequence 2,3…,19. That is,

$$\tau_{2,t} = \left\{ \begin{array}{cl} t & \text{for } t = 1,2,\dots,g_{1}-1\\ t+1 & \text{for } t = g_{1}, g_{1}+1,\dots,g_{2}-2\\ t+2 & \text{for } t = g_{2}-1,g_{2},\dots,17, \end{array} \right. $$

as represented by the conditional independence graph in Fig. 2. Note that due to the experimental set-up, we constrain the first expression values from Willunga and Clare to align (*τ*_1,1_=*τ*_2,1_=1). The log-likelihood of the alignment model for the grapevine data is then

(1) $$ {\small{\begin{aligned} \ell({\lambda}, g_{1},g_{2}|\boldsymbol{w},\boldsymbol{c}) &= \sum_{k=1}^{8644}\log \left[\sum_{(s_{1},s_{2},\ldots,s_{T})} p\left(s_{1},s_{2},\ldots,s_{T}\right)\right.\\ &\quad\times \!\prod_{t=1}^{19} p\left(w^{(k)}_{t}\big|S_{\tau_{1,t}}=s_{\tau_{1,t}}\right) \\&\quad\left.\times\prod_{t=1}^{17} p\left(c^{(k)}_{t}\big|S_{\tau_{2,t}}=s_{\tau_{2,t}}\vphantom{c^{(k)}_{t}\big|S_{\tau_{2,t}}}\right) {\vphantom{\prod_{t=1}^{17}}}\right]. \end{aligned}}}  $$

There are well established methods for efficient calculation of the likelihood, finding the Viterbi paths and estimating the model parameters for a standard HMM [11]. These methods are readily adapted to our alignment model defined by (1) if the gaps *g*_1_ and *g*_2_ are given. Note that our alignment HMM is a special case of a hidden semi-Markov model [12].

### Alignment model fitting method

We fit the alignment HMM to the grapevine data by maximising the log-likelihood *ℓ*(*λ*,*g*_1_,*g*_2_) with respect to the HMM parameters *λ* and the gap positions *g*_1_ and *g*_2_. A profile likelihood approach could be implemented by applying the Baum-Welch algorithm [11] to obtain an estimate $\hat \lambda ^{*}(g_{1},g_{2})$ for the HMM parameters for each pair (*g*_1_,*g*_2_) and then maximising the profile likelihood $\ell (\hat \lambda ^{*}(g_{1},g_{2}),g_{1},g_{2})$ with respect to *g*_1_ and *g*_2_.

We propose a two-step approach with a much lower computational requirement and greater robustness to non-aligned expression profiles. In the first step, an estimate $\hat \lambda $ for the HMM parameter is obtained, independent of the pairing and of the gap positions. In the second step, the log-likelihood $\ell (\hat \lambda,g_{1},g_{2})$ is evaluated for each pair (*g*_1_,*g*_2_) and the maximum likelihood estimates are selected from the enumeration. The estimate $\hat \lambda $ is obtained from modelling each individual expression profile at both Willunga and Clare by a standard HMM [11] in which the same parameters *λ* apply to both vineyards. Such a model is implied by (1) when dropping the constraint that each pair of emission sequences correspond to a common state sequence.

The computational advantage of this approach is that it requires only a single maximisation of the HMM likelihood rather than one for each pair of gap positions. More importantly, it is also robust against the influence of expression profiles not suitable for alignment. The notion of a common alignment is plausible for developmental genes but not for those driven by environmental factors such as temperature. Since the non-developmental genes are not known in advance, they cannot be removed and their presence may produce significant bias in the estimate $\hat \lambda ^{*}(\hat g_{1},\hat g_{2})$. A minor issue is that the standard HMM model from which $\hat \lambda $ is obtained is inconsistent with the alignment HMM (1) because of the gaps in the Clare sequence. However, it is reasonable to assume that any bias arising from this inconsistency is minor compared to that arising from non-aligned expression profiles in the full likelihood estimate $\hat \lambda ^{*}(\hat g_{1},\hat g_{2})$.

To summarise, we produce an alignment for the grapevine data in the following steps:

1. The gene expression profiles are filtered so that only those with significant differential expression and at least 2-fold change in expression over the time course are retained.
2. Each expression profile is linearly rescaled to lie in the interval [0,1].
3. A standard HMM is fitted to the data to obtain the estimated HMM parameters $\hat \lambda $.
4. The gap positions are estimated by maximising the alignment HMM log-likelihood $\ell (\hat \lambda,g_{1},g_{2})$ with respect to *g*_1_ and *g*_2_.
5. A single representation of the aligned expression profiles can be obtained either by averaging the aligned expression profiles or by finding the Viterbi path.

We implemented our methodology in MATLAB by adapting the code provided in the HMM Toolbox [13].

## Results and discussion

**Fig. 3:**
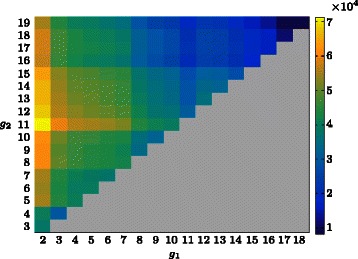
Heat-map of the alignment HMM log-likelihood for the grapevine data (1) evaluated using $\hat {\lambda }$ and each possible combination of the gap positions 1<*g*_1_<*g*_2_≤19

A standard HMM with *N*=5 states was fitted to the grapevine data. The variances of the emission distributions were constrained so that ${\sigma _{j}^{2}} \geq 0.001$ for *j*=1,2,…,5. This constraint was applied to avoid difficulties arising from the fact that the distribution of scaled expression values has point masses at the endpoints 0 and 1 (Additional file 1: Figure S1). The gap positions that maximise the log-likelihood $\ell (\hat \lambda,g_{1},g_{2})$ were found to be $\hat {g}_{1} = 2$ and $\hat {g}_{2} = 11$ (Fig. 3). The single peak in Fig. 3 indicates that the gap positions are well determined for the grapevine data.

**Fig. 4:**
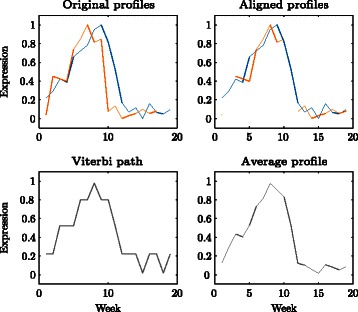
Expression profiles from the Willunga (blue) and Clare (orange) vineyards, aligned expression profiles, Viterbi path and average profile representation for gene 1621649_at. The Viterbi path is plotted at the estimated means of the emission distributions

**Fig. 5:**
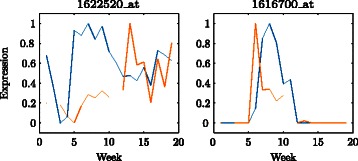
Poorly aligned expression profiles for two example genes from the Willunga (blue) and Clare (orange) vineyards

Figure 4 shows the aligned expression profile for gene 1621649_at, together with the Viterbi path and average profile representations. For this gene, the alignment HMM has produced a suitable alignment. The method performs similarly for the other genes shown in Fig. 1. On the other hand, Fig. 5 shows poorly aligned expression profiles for genes 1622520_at and 1616700_at. For gene 1622520_at, the expression profiles at Willunga and Clare have very different shapes and cannot be aligned. The expression profiles for gene 1616700_at have similar shapes at Willunga and Clare but are not well aligned by the estimated gaps $\hat g_{1}=2$ and $\hat g_{2}=11$.

**Fig. 6:**
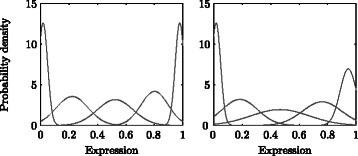
Estimated emission densities corresponding to $\hat {\lambda }$ (left) and $\hat {\lambda }^{*}(\hat g_{1},\hat g_{2})$ (right)

For the purpose of comparison, the parameters *λ* were also estimated from the alignment HMM (1) with fixed gaps $\hat {g_{1}} = 2$ and $\hat {g_{2}} = 11$. The estimated emission distributions for $\hat \lambda $ and $\hat \lambda ^{*}(\hat g_{1},\hat g_{2})$ are shown in Fig. 6. In both cases the estimated means are spaced evenly across the range [0,1]. However, for $\hat \lambda ^{*}(\hat g_{1},\hat g_{2})$, the estimated variances are noticeably larger. An explanation for this difference is the presence of genes with expression profiles that are not suitable for alignment. In particular, the presence of misaligned profiles will lead to very different expression values being aligned at the same time point and equally contributing to the parameter estimates for the same state, hence inflating the estimated variance.

We consider the robustness of the estimates of the gap positions. In a simulation experiment, even with up to 80 % of the data not suitable for alignment, the true gaps can clearly still be found through the log-likelihood (Additional file 2: Figure S2). For subsets of simulated profiles with different true gap positions, the maximum peak in the log-likelihood heat-map becomes less concentrated and spreads out (Additional file 2: Figure S2). For the grapevine data, the log-likelihood is sharply peaked (Fig. 3) and the estimated gaps additionally conform with other physiological features measured on the berries during the experiment. For example, both total soluble solids (sugar content) and berry weight were also measured weekly at Willunga and Clare and the same gap positions appear to work well for this additional data (Additional file 3: Figure S3).

We also consider fitting the alignment model with different choices of the number of states *N*. The estimated emission densities and heat-maps for *N*=3 and *N*=7 are given in Additional file 4: Figure S4. We can see that the same maximum likelihood gaps are found in both cases. It appears that *N*=3 states is not enough over the range of the data while *N*=7 is too many as two of the emission densities coincide.

It is the difference between the estimates $\hat \lambda $ and $\hat \lambda ^{*}(\hat g_{1},\hat g_{2})$ seen in Fig. 6 that suggests the presence of poorly aligned profiles in the grapevine data. To identify the well and poorly aligned expression profiles we consider the Hamming distance between the Viterbi path for each pair of aligned profiles and the Viterbi paths obtained for the individual profiles. Let $\hat {S}^{(k)}_{1:19}$ be the alignment HMM Viterbi path for the *k* ^*th*^ pair of profiles, and let $\hat Sw^{(k)}_{1:19}$ and $\hat S{c}^{(k)}_{1:17}$ be the standard HMM Viterbi paths for the *k* ^*th*^ Willunga and Clare profiles respectively. The Hamming distance between the Viterbi paths for the *k* ^*th*^ pair of expression profiles is

$$\begin{array}{*{20}l} H(k) = \sum_{t = 1}^{19} I\left\{\hat{S}^{(k)}_{\tau_{1,t}} \neq \hat S{w}^{(k)}_{t} \right\} + \sum_{t = 1}^{17} I\left\{\hat{S}^{(k)}_{\tau_{2,t}} \neq \hat S{c}^{(k)}_{t} \right\}. \end{array} $$

The Hamming distance *H*(*k*) has a negative linear relationship to log-likelihood (Additional file 5: Figure S5). Table 1 shows the Hamming distances and the log-likelihoods for the example expression profiles shown in Figs. 1 and 5. Well aligned expression profiles typically have high log-likelihood and low Hamming distance while conversely, the poorly aligned expression profiles typically have low log-likelihood and high Hamming distance. Not all profiles are obviously well or poorly aligned. Note that the aligned profiles for gene 1622520_at have relatively high log-likelihood because they are well aligned for all the time points when the gene exhibits low expression (Fig. 5). While the Hamming distance is purely a measure of the quality of alignment as determined by the Viterbi paths, the log-likelihood incorporates other aspects of model fit such as the distance from the expression values to the state means. For this reason we recommend the Hamming distance to identify poorly aligned expression profiles.

**Table 1:** Log-likelihood and Hamming distance for the example pairs of profiles given in Figs. 1 and 5

Affy ID	Figure	Log-likelihood	*H*(*k*)
1621649_at	Fig. 1	17.4670	7
1610245_at	Fig. 1	41.7735	6
1616418_at	Fig. 1	25.6842	7
1609985_at	Fig. 1	18.3318	10
1622520_at	Fig. 5	-43.0573	18
1616700_at	Fig. 5	24.1855	10

As previously outlined, how well a pair of expression profiles align across vineyards is evidence for whether the corresponding gene is likely to be developmentally driven. To illustrate the potential for identification of biological function from alignment, a set of 198 genes were considered as test data (Additional file 6). Although this test data were also used to train the alignment model, we never made use of the labels in the model fitting. Our classifier arises out of the diagnostics of the alignment HMM as we assume there is a correspondence between ‘well aligned’ and ‘developmental’.

**Fig. 7:**
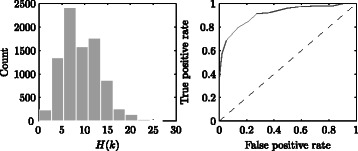
Histogram of *H*(*k*) for the grapevine data (left) and ROC curve for classifying ‘developmental’ or ‘non-developmental’ (temperature responsive) genes based on whether they are above or below a given *H*(*k*) threshold (right)

The left side of Fig. 7 shows the distribution of Hamming distances for all pairs of expression profiles in the grapevine data. The right side of Fig. 7 shows the receiver operating characteristic (ROC) curve for classifying genes as ‘developmental’ or ‘non-developmental’ (temperature responsive) based on whether the Hamming distance is below or above a given threshold. The area under the curve is 0.91, indicating a good level of discrimination for this data. When the threshold is taken as *H*(*k*)=10, the true positive rate is 85.3 *%* and the false positive rate is 21.9 *%*. This suggests that applying the same threshold is a potentially useful filter for the classification of developmentally controlled genes amongst a set of genes of unknown function.

Grimplet *et al.* [3] surveyed the current gene function annotation for grapevines. Assigning a developmental role to genes based on the putative function of the proteins they encode, as determined by sequence similarity to other genes of known function and without reference to their expression patterns, is an uncertain practice. For example, so-called ‘heat shock’ proteins with similar protein sequences may be either developmentally controlled or may be induced by changes in temperature, or both. Additionally, differences in the promoter sequences of genes encoding similar proteins may determine whether a gene is involved in a developmentally controlled process or not.

By comparing the expression of genes under different growth conditions, as has been done in this paper, we are able to gain evidence regarding the reproducibility of gene expression patterns indicative of a role in development as opposed to a response to external signals. This information can be used as additional evidence in the further investigation of gene function. For example, using the annotation of Grimplet *et al.* [3], of the 8644 genes represented in the grapevine data, 1968 have no description of possible function (‘no function’, ‘no hit’, ‘unknown’ or ‘unknown function’) and of these we find 1279 probe sets with *H*(*k*)≤ 10. That is, 1279 genes with no current annotated function are well aligned between the Willunga and Clare vineyards and therefore we now have additional information that these genes are likely to be controlled in a developmental manner Additional file 7.

The proposed alignment method could be extended and refined in a number of ways. In particular, potential improvements may be obtained through more detailed modelling of the emission distributions in the HMM. In the present paper, we have applied Gaussian emission distributions to the expression profiles averaged over replicates within vineyards. This approach could be refined by considering the replicates as multivariate observations instead of averaging and also by considering alternatives to the Gaussian emission assumption. Autoregressive emissions as well as higher-order Markov components of HMMs have been investigated and found to improve performance in the identification of over-expressed genes [14]. The incorporation of this structure into our framework may more realistically model the expression profiles with potential improvements in performance. The implementation and evaluation of these improvements are the subject of future research.

## Conclusion

We have presented a novel alignment method based on an HMM and demonstrated the alignment on the grapevine data. This is a model suitable for sparse time course data where interpolation is not appropriate. The estimated model parameters have simple interpretations and the estimated gap positions are well determined for the grapevine data. We have demonstrated that the estimates of the HMM parameters as well as the gap positions are robust against subsets of profiles that are not suitable for alignment. For pairs of expression profiles that are well aligned, the Viterbi paths or average profile representations can be used as the input to downstream analysis of the data. This allows for an integrated analysis of multiple site time course gene expression data such as the Willunga and Clare grapevine data.

We have demonstrated the use of the Hamming distance and the log-likelihood as a measure of quality for the alignment of a pair of expression profiles. Pairs of profiles that are well aligned will have high log-likelihood and a small Hamming distance while the poorly aligned pairs will have low log-likelihood and a large Hamming distance. We have also shown, for a set of genes with known function, that classification of genes according to the Hamming distance has reasonable predictive power for the classification of developmentally driven genes. This both validates that the alignment we obtain is meaningful and also suggests the potential for helping to identify the role of genes with unknown function.

## Availability of supporting data

The MATLAB code and grapevine data to obtain all of the output described in this paper are provided as Additional files. The raw gene expression data is stored at NCBI in the GEO database as GSE7677 (Willunga) and GSE8445 (Clare) Additional file 8.

## Additional files

Additional file 1
**Figure S1.** Histogram of the scaled expression levels for the grapevine data overlaid with a mixture density of the estimated emission densities where the mixture coefficients are the stationary Markov transitions of the estimated alignment HMM parameters $\hat {\lambda }$.

Additional file 2
**Figure S2.** Heat-maps corresponding to a number of simulation experiments. Top row: 1000 pairs of profiles were simulated using the estimated HMM parameters $\hat {\lambda }$ and with true gap positions (*g*_1_=5,*g*_2_=13). Pairs of profiles not suitable for alignment were obtained by permuting the pairing information of a subset of profiles. From left to right: Heat-maps calculated using the simulated data and parameters $\hat {\lambda }$ with an increasingly large subset of profiles not suitable for alignment. Middle row: Same simulation set-up with true gaps of either (5, 13) or (8, 16). From left to right: Heat-maps calculated using the simulated data and parameters $\hat {\lambda }$ with an increasingly mixed proportion of pairs of profiles with different true gaps. Bottom row: Same simulation set-up with true gaps of either (4, 9) or (12, 17).

Additional file 3
**Figure S3.** Total soluble solids (left) and average berry weight (right) measured over the development cycle at the Willunga (blue) and Clare (orange) vineyards with the same alignment as found for the grapevine expression data. Note that these measurements did not commence at the beginning of the experiment.

Additional file 4
**Figure S4.** Estimated emission densities and heat-maps when fitting the alignment model with *N*=3 (top) and *N*=7 (bottom) states to the grapevine data.

Additional file 5
**Figure S5.** Log-likelihood under the alignment HMM by Hamming distance for each pair of expression profiles in the grapevine data.

Additional file 6
**The set of 198 labelled genes (test data).** From a separate experiment, 96 of these genes had been identified as ‘temperature responsive’ genes through the response of gene expression to changes in temperature. The remaining 102 genes were selected from the Grapevine Affymetrix array probe list on the basis of annotated function where selected genes were thought to be involved in a developmental process in grapevine (and often in other plant species) and, where possible, on the basis of gene expression patterns throughout development.

Additional file 7
**Final grapevine output.** Log-likelihood under the alignment HMM, Hamming distance and current annotation [3] for all 8644 genes in the grapevine data.

Additional file 8
**MATLAB code and grapevine data.** The MATLAB code and grapevine data to obtain all of the output described in this paper.

## References

[1] Aach J, Church GM (2001). Aligning gene expression time series with time warping algorithms. Bioinformatics.

[2] Pearce I, Coombe BG, Dry PR, Coombe BG (2004). Grapevine phenology. Viticulture. Volume 1 - Resources.

[3] Grimplet J, Van Hemert J, Carbonell-Bejerano P, Díaz-Riquelme J, Dickerson J, Fennell A (2012). Comparative analysis of grapevine whole-genome gene predictions, functional annotation, categorization and integration of the predicted gene sequences. BMC Res Notes.

[4] Lin T, Kaminski N, Bar-Joseph Z (2008). Alignment and classification of time series gene expression in clinical studies. Bioinformatics.

[5] Schliep A, Costa IG, Steinhoff C, Schönhuth A (2005). Analyzing gene expression time-courses. IEEE/ACM Trans Comput Biol Bioinform.

[6] Durbin R, Eddy S, Krogh A, Mitchison G (1998). Biological sequence analysis.

[7] Yuan M, Kendziorski C (2006). Hidden Markov models for microarray time course data in multiple conditions. J Am Stat Assoc.

[8] Yoneya T, Mamitsuka H (2007). A hidden Markov model-based approach for identifying timing differences in gene expression under different experimental factors. Bioinformatics.

[9] Listgarten J, Neal RM, Roweis ST, Emili A (2004). Multiple alignment of continuous time series. Adv Neural Inf Process Syst.

[10] Smyth GK (2004). Linear models and empirical Bayes methods for assessing differential expression in microarray experiments. Stat Appl Genet Molec Biol.

[11] Rabiner LR (1989). A tutorial on hidden Markov models and selected applications in speech recognition. Proc IEEE.

[12] Yu SZ (2010). Hidden semi-Markov models. Artif Intell.

[13] Murphy K. The HMM Toolbox. http://www.cs.ubc.ca/~murphyk/Software/HMM/hmm.html. Accessed 7 December 2014.

[14] Seifert M, Abou-El-Ardat K, Friedrich B, Klink B, Deutsch A (2014). Autoregressive higher-order hidden Markov models: exploiting local chromosomal dependencies in the analysis of tumor expression profiles. PLOS One.

