# Supplementary figures and images for: Alignment of time course gene expression data and the classification of developmentally driven genes with hidden Markov models
Source: BMC Bioinformatics. 2015 Jun 18;16:196. doi: 10.1186/s12859-015-0634-9 (PMC4472167; doi:10.1186/s12859-015-0634-9)

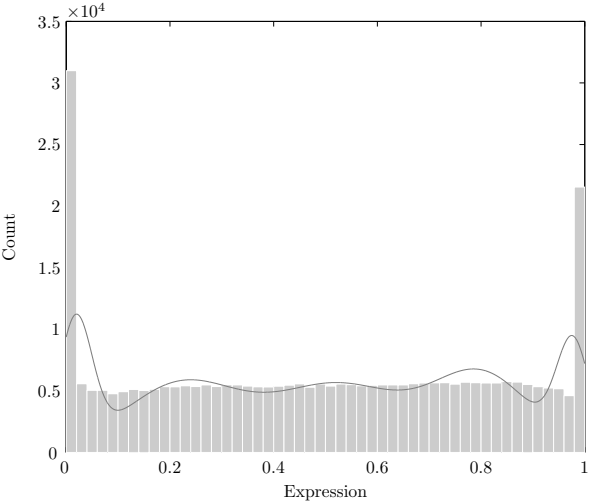

Supplement: Additional file 1 — Figure S1. Histogram of the scaled expression levels for the grapevine data overlaid with a mixture density of the estimated emission densities where the mixture coefficients are the stationary Markov transitions of the estimated alignment HMM parameters $\hat {\lambda }$λ^. [file 12859_2015_634_MOESM1_ESM.pdf]

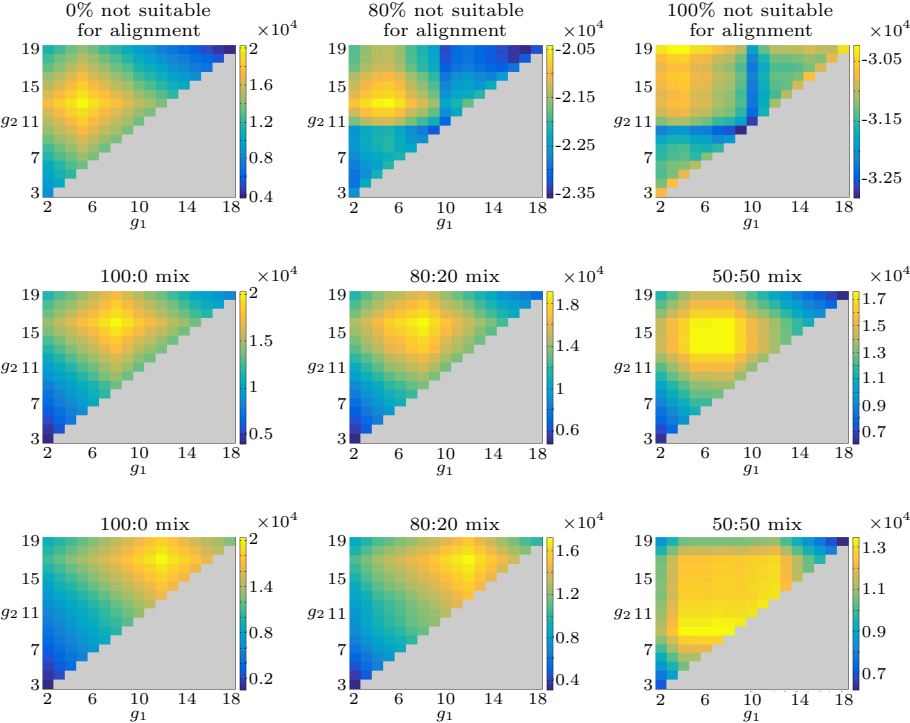

Supplement: Additional file 2 — Figure S2. Heat-maps corresponding to a number of simulation experiments. Top row: 1000 pairs of profiles were simulated using the estimated HMM parameters $\hat {\lambda }$λ^ and with true gap positions (g 1=5,g 2=13). Pairs of profiles not suitable for alignment were obtained by permuting the pairing information of a subset of profiles. From left to right: Heat-maps calculated using the simulated data and parameters $\hat {\lambda }$λ^ with an increasingly large subset of profiles not suitable for alignment. Middle row: Same simulation set-up with true gaps of either (5, 13) or (8, 16). From left to right: Heat-maps calculated using the simulated data and parameters $\hat {\lambda }$λ^ with an increasingly mixed proportion of pairs of profiles with different true gaps. Bottom row: Same simulation set-up with true gaps of either (4, 9) or (12, 17). [file 12859_2015_634_MOESM2_ESM.pdf]

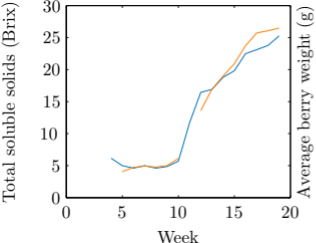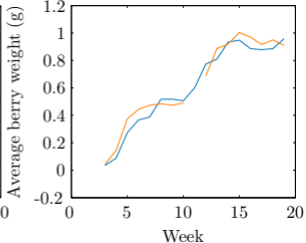

Supplement: Additional file 3 — Figure S3. Total soluble solids (left) and average berry weight (right) measured over the development cycle at the Willunga (blue) and Clare (orange) vineyards with the same alignment as found for the grapevine expression data. Note that these measurements did not commence at the beginning of the experiment. [file 12859_2015_634_MOESM3_ESM.pdf]

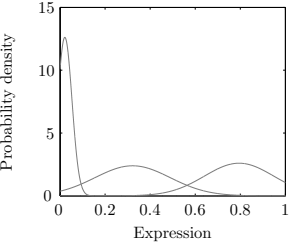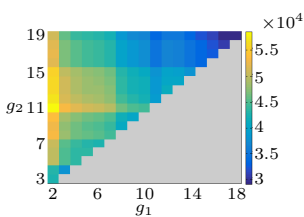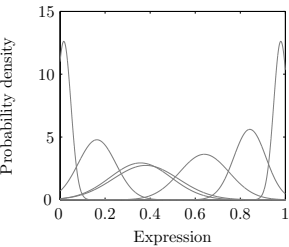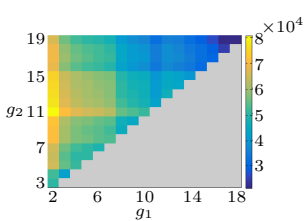

Supplement: Additional file 4 — Figure S4. Estimated emission densities and heat-maps when fitting the alignment model with N=3 (top) and N=7 (bottom) states to the grapevine data. [file 12859_2015_634_MOESM4_ESM.pdf]

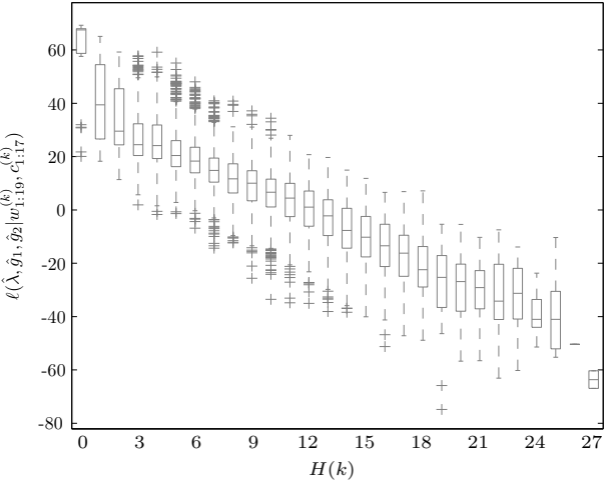

Supplement: Additional file 5 — Figure S5. Log-likelihood under the alignment HMM by Hamming distance for each pair of expression profiles in the grapevine data. [file 12859_2015_634_MOESM5_ESM.pdf]
